# Serum Vitamin D in Patients with Chronic Obstructive Lung Disease Does Not Correlate with Mortality – Results from a 10-Year Prospective Cohort Study

**DOI:** 10.1371/journal.pone.0053670

**Published:** 2013-01-14

**Authors:** Dennis Back Holmgaard, Lone Hagens Mygind, Ingrid Louise Titlestad, Hanne Madsen, Palle Bach Nielsen Fruekilde, Svend Stenvang Pedersen, C. Pedersen

**Affiliations:** 1 Department of Infectious Diseases Q, Odense University Hospital, Odense, Denmark; 2 Department of Infectious Diseases, Aalborg Hospital, Aalborg, Denmark; 3 Department of Respiratory Medicine J, Odense University Hospital, Odense, Denmark; 4 Department of Clinical Biochemistry and Pharmacology, Odense University Hospital, Odense, Denmark; University of California San Francisco, United States of America

## Abstract

**Background:**

Recent studies have found vitamin D (25-OHD) deficiency and insufficiency to be common among patients with COPD. Serum level of 25-OHD seems to correlate to pulmonary function, COPD disease staging, and increased susceptibility to respiratory infections. We wanted to investigate whether vitamin D deficiency or insufficiency was associated with mortality rate in patients suffering from advanced COPD.

**Methods:**

25-OHD serum levels were measured in 462 patients suffering from moderate to very severe COPD. Patients were stratified into three groups according to serum levels of 25-OHD. Outcome measure was mortality in a 10 year follow-up period. Kaplan-Meier curves (KM) were plotted and mortality hazard ratios (HR) were calculated using Cox Proportional Hazard regression (Cox PH).

**Results:**

Serum 25-OHD deficiency and insufficiency were prevalent. We were unable to demonstrate any association between baseline serum levels of 25-OHD and mortality rate. We found an association between mortality and age [HR 1.05 (CI 95%: 1.03–1.06)], Charlson score [HR 1.49 (CI 95%: 1.06–2.09)], increasing neutrophil count [HR 1.05 (CI 95%: 1.02–1.09)], severe [HR 1.41 (CI 95%: 1.06–1.86)]/very severe COPD [HR 2.19 (CI 95%: 1.58–3.02)] and a smoking history of more than 40 pack years [HR 1.27 (CI 95%: 1.02–1.70)].

**Conclusions:**

Serum level of 25-OHD does not seem to be associated with mortality rate, suggesting no or only a minor role of 25-OHD in disease progression in patients with moderate to very severe COPD.

## Introduction

Chronic obstructive pulmonary disease (COPD) is characterized by an irreversible loss of airflow, probably due to airway destruction caused by inflammatory responses to airway irritants and noxious gases like those found in tobacco smoke [1]. The disease is usually progressive and COPD is a leading cause of mortality, currently the fifth leading cause of mortality in the world [2]–[4]. The rate of progression in terms of decline in pulmonary function is subject to a high degree of individual variation the nature of which have been the focus of several studies [5]–[8].

The role of vitamin D in calcium homeostasis is well established. Vitamin D can be obtained from several sources, but the predominant source of vitamin D in humans is UVB radiation (290–315 nm) [9]. The primary storage form is 25-hydroxyvitamin D (25-OHD). Final hydroxylation to the active form 1,25-dihydroxyvitamin primarily takes place in the kidneys, but several other tissues have been shown to exhibit 1α-hydroxylase activity, e.g. lung epithelia [10], dendritic cells [11], monocytes [12] and activated B cells [13] suggesting a possible role for localized tissue production of vitamin D.

Vitamin D has been recognized as a mediator of the immune system *in vitro* for more than 25 years [14], [15], and many studies indicate that vitamin D may be a key player in immune regulation [16], autoimmune diseases [17] and in the development of cancer [18]. Vitamin D inhibits metalloproteinase [19], inhibits fibroblast proliferation and is involved in collagen synthesis suggesting a role in tissue remodulation [20]. In addition to this, studies have shown an association between respiratory infections and low levels of vitamin D [21], [22].

25-OHD serum levels in the range 20–30 ng/ml is considered as 25-OHD insufficiency, and levels below 20 ng/ml as deficiency [23]. Both insufficiency and deficiency have been shown to be very common among patients with COPD [24]. This is not surprising as most COPD patients are at an increased risk of having low vitamin D blood levels due to reduced outdoor activity, old age, accelerated skin ageing, and treatment with glucocorticoids that induce vitamin D catabolism. Because of this, it has been argued that low vitamin D blood level is just an expected consequence of COPD progression. Prior studies have found a significant correlation between pulmonary function and vitamin D serum levels [25], and a correlation between COPD stage and 25-OHD serum levels [24]. The authors of these studies argue for a possible role of vitamin D in pulmonary health. Other studies found no correlations between 25-OHD plasma levels and the rate of decline in lung function [6], or the risk of developing acute exacerbations of COPD [26]. Recently a 1 year intervention trial found no effect of high dose vitamin D supplementation on mortality, but in a post-hoc analysis of 30 patients there was an effect on exacerbation frequency [27]. Thus the long term effects of 25-OHD deficiency/insufficiency in COPD and its role in pulmonary health and mortality remains largely undetermined.

In this study we sought to elucidate the connection between COPD, vitamin D blood levels and its relation to long-term mortality.

## Methods

### Study Participants

Study participants were patients who were originally included in a randomized controlled trial (RCT) investigating the effects of administering prophylactic Azithromycin in 3 doses of 500 mg every month. Primary outcome was change in post-bronchodilator FEV_1_. Secondary outcomes included number of hospital admissions, number of hospital days, mortality, quality of life, use of medication, prevalence of respiratory pathogens and prevalence of macrolide resistance. Inclusion criteria included: Age above 50 years, with a history of at least one prior admission to hospital for COPD in exacerbation within the last two years; post-bronchodilator spirometry with a FEV1<60% (4 weeks after hospitalization) and <300 ml FEV1 reversibility. In addition to this patients were only included if deemed in a stable phase of their disease, defined as having no usage of antibiotics up until 1 week before inclusion to the study and with no admissions to hospital within the last month before inclusion.

Exclusion criteria included: Patients with known other respiratory tract infection, e.g. tuberculosis or aspergillosis; pulmonary malignancy; other pulmonary diseases than COPD; known hereditary disposition to lung infections such as alfa-1-antitrypsin deficiency, cystic fibrosis or primary ciliary dyskinesia; receiving long-term antibiotic treatment (e.g. recurrent cystitis); known allergy or intolerance to azithromycin; pregnant or breastfeeding women; manifest heart, liver or renal insufficiency; patients that, for reasons not stated above, were unlikely to be able to participate in a study period of 3 years.

The patients were followed for 36 months, and were seen in the out-patient clinic every 3^rd^ month. The study did not demonstrate an effect of Azithromycin on long-term mortality. A more comprehensive description of the study, including criteria for inclusion/exclusion, is accessible at http://clinicaltrials.gov - identifier NCT00132860 (accessed November 29^th^ 2012).

### Serum Samples

Four hundred sixty two serum samples were taken at enrollment centrifuged, aliquoted and stored at −80°C until analysis. Samples were measured for serum 25-OHD using liquid chromatography tandem mass spectrometry (LC-MS/MS). The samples were analyzed at Odense University Hospital – Dept. of Clinical Biochemistry and Pharmacology, a DEQAS (vitamin D External Quality Assessment Scheme) certified laboratory – for details see http://www.deqas.org (accessed November 29^th^ - 2012). All samples, and all steps of the analysis were performed by a single experienced technician. All samples were performed in duplicates. CV was <7.5% at 25-OHD serum levels above 10 nmol/l (4.12 ng/ml).

Ethical permission for the study was obtained from the The Regional Scientific Ethical Committee for Southern Denmark approval number VF 19990031.

### Statistics

Study participants were stratified into 3 groups according to serum levels of 25-OHD, >30 ng/ml, 30–20 ng/ml and <20 ng/ml. These limits are generally used to define vitamin D insufficiency and deficiency [23]. Kruskal-Wallis test for equality-of-populations rank test or Chi-squared test was used, as appropriate, to compare distribution of variables between different levels of serum 25-OHD at baseline.

We collected patient person-years of follow-up for all patients from inclusion in the study (May 2001 to April 2004) until date of death, emigration, lost to follow up or we had passed January 31^st^ 2011, whichever came first. Patient date of death was registered in Danish central CPR registry and was collected up until January 31^st^ 2011. Follow-up was 99.1% complete at study completion January 31^st^ 2011.

Kaplan Meier (KM) curves were computed to illustrate the time to death in the three groups of serum 25-OHD. We determined to use a Cox Proportional Hazard (Cox PH) analysis to calculate hazard rates (HR) and 95% confidence intervals (95% CI). Our primary variable was 25-OHD serum level. We primarily decided to include BMI <20, COPD stage [28], Charlson score (>3 or higher), age (as a continuous variable), treatment group (Azithromycin vs. placebo) as possible confounders. In addition to this, a potential panel of confounders was tested in univariate analysis, and they were included in the final model if they were significant at a level of 0.25 or below. Neutrophils and pack-years >40 were included in this way. Active smoking status at baseline, and CRP were excluded from the analysis for the same reasons. Variables were tested for interaction when deemed of clinical relevance; by comparing models with and without interaction terms using likelihood ratio test statistics. No significant interactions were found. The 462 patients with 353 fatalities provided our study with power to demonstrate a coefficient difference of 0.3 (HR 1.35) with a power of 80%.

Proportional hazard assumptions were tested for all categorical variables using log-log plots and expected vs. observed plot. Continual variables were tested using observed vs. expected variables and were tested for the linearity assumption. The collected dataset was then tested using a global test using Schöenfeldt’s Residuals which rejected a potential deviation from the proportional Hazard assumption (p = 0.28).

Differences in 25-OHD serum based on seasonal variation were displayed using Box-plots and tested for significance using Wilcoxon Rank sum test. Winter was defined as the period from December to February, spring as March to May, summer as June to August and fall as September to November. All time periods included three months.

A scatter plot displaying FEV1% pred vs. serum 25-OHD was computed. Strength of correlation between FEV1 (% pred) and serum 25-OHD was determined using Spearman’s correlation rank coefficient.

Results in all tables are presented as median with 95% confidence interval (CI) or interquartile range or rates as appropriate. All statistical analyses were carried out using STATA 11.1 (Stata Corp LP, TX, USA).

## Results

Out of 574 participants in the main trial, 462 had serum samples available for this secondary analysis (230 men and 232 women). The cohort was characterized by low median serum levels of 25-OHD (mean 22.38 [SD 10.00]). Baseline characteristics of the study population in relation to serum 25-OHD levels showed that lower levels of neutrophils were associated with low levels of serum 25-OHD. A high proportion of smokers at baseline and a higher BMI were associated with lower serum 25-OHD levels. The population in general was characterized by having advanced COPD as indicated by very low median values of FEV1% predicted. BMI values were within normal range across all 4 groups, i.e. between 18.5 and 25 as defined by the WHO.(http://apps.who.int/bmi/index.jsp?introPage=intro_3.html) – accessed September 29^th^- 2012).

The patient group that was not included into the study due to lack of eligible serum samples did not differ in demographics compared to any of the examined groups (Table 1– column 4).

**Table 1:** Distribution of baseline characteristics based on conventional 25-OHD levels.

	Serum 25-OHD<20 ng/ml(n = 207)	Serum 25-OHD20–30 ng/ml(n = 141)	Serum 25-OHD>30 ng/ml(n = 114)	Patients notIncluded*(n = 112)
**Age at index date** **(years) Median (IQR)**	70(64–75)	71(65–76)	71(66–77)	70.5(66–75)
**Charlsonscore** **Median (IQR)**	2(1–2)	2(1–3)	2(1–3)	2(1–3)
**Smoking** **(years) Median (IQR)**	38.5(27–51)	38.5(27–53.5)	38(25–49)	39.5(25–50)
**Neutrophils (×10∧9/L)** **Median (IQR)** **	6.08(4.42–7.28)	6.35(4.77–8.94)	6.30(4.93–9.46)	6.87(4.98–8.78)
**FEV_1_ (L)** **Median (IQR)**	0.93(0.73–1.17)	0.87(0.69–1.1)	0.85(0.64–1.12)	0.88(0.71–1.18)
**FEV1 predicted (%)** **Median (IQR)**	40.38(31.72–48.15)	36.87(30.00–47.94)	38.45(29.73–48.21)	37.46(30.37–44.68)
**FVC (L)** **Median (IQR)**	2.04(1.59–2.48)	2.00(1.50–2.40)	1.85(1.43–2.32)	2.04(1.62–2.44)
**BMI** ** **Median (IQR)**	24.57(20.69–28.58)	24.31(21.45–27.56)	23.16(20.15–26.12)	23.95(21.03–27.57)
**Present smokers** **n (%)** ***	97(46.63)	43(30.50)	40(35.09)	44(43.14)
**Male gender** **n (% )**	108(52.17)	71(50.35)	51(44.74)	59(57.84)
**Randomization** **Azithromycin n (%)**	102(49.28)	77(54.61)	57(50.00)	48(47.06)

&ast; Patients without eligible serum samples.

&ast;&ast; Significant difference between groups using Kruskal-Wallis equality-of-populations rank test.

&ast;&ast;&ast; Significant difference between groups using X^2^ test.

**Figure 1:**
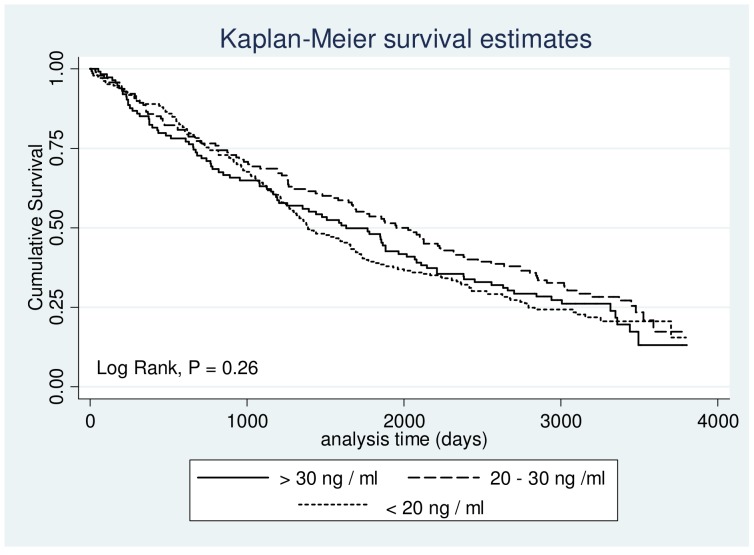
Three-year survival according to conventional levels of serum 25-OHD deficiency and insufficiency. Differences between groups were examined using a log-rank test. Result is displayed as a p value in the lower left corner.

**Figure 2:**
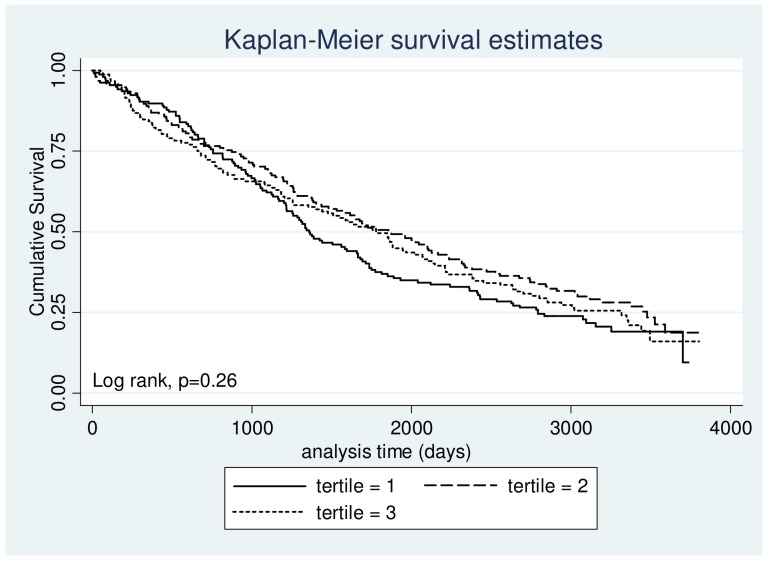
Three-year survival according to levels of serum 25-OHD distributed on tertiles. Differences between groups were examined using a log-rank. Result is displayed as a p value in the lower left corner.

We were not able to demonstrate any association between mortality and the three predefined strata of 25-OHD serum levels (figure 1). Findings were similar when we did a post hoc analysis dividing patients into 3 equally large groups based on 25-OHD serum levels (figure 2). Mortality was very high, and about three quarters of the study population died during the observation period, i.e. 353 patients of 462 (76.41%).

To adjust for baseline prognostic factors, a multivariate Cox regression was performed. As shown in table 2 there was no significant difference in mortality in relation to serum level of 25-OHD, i.e. 20–30 ng/ml [HR 0.90 (CI 95%: 0.67–1.21)]and <20 ng/ml [HR 1.11 (CI 95%: 0.85–1.46)]. The results display a significant correlation between mortality and age [HR 1.05 (CI 95%: 1.03–1.06)], Charlson score >3 [HR 1.49 (CI 95%: 1.06–2.09)], increasing neutrophil count [HR 1.05 (CI 95%: 1.02–1.09)], severe [HR 1.41 (CI 95%: 1.06–1.86)]/very severe COPD [HR 2.19 (CI 95%: 1.58–3.02)] and a smoking history of more than 40 pack years [HR 1.27 (CI 95%: 1.02–1.70)] at baseline.

**Table 2:** Uni- and multivariate Proportional Hazard Cox Regression of prognostic markers for mortality.

	UnadjustedHazard Ratio(95% CI)	P-value	AdjustedHazard ratio(95% CI)	P-value
**Serum 25-OHD** **>30 ng/ml**	Ref. Value	–	Ref. Value	–
**Serum 25-OHD** **20–30 ng/ml**	0.84(0.63–1.12)	0.234	0.90(0.67–1.21)	0.48
**Serum 25-OHD** **<20 ng/ml**	1.03(0.79–1.33)	0.83	1.11(0.85–1.46)	0.45
**Age pr. Year** **above age 50** *	1.04(1.03–1.06)	<0.001	1.05(1.03–1.06)	<0.001
**Charlsonscore** **>3**	1.53(1.11–2.11)	0.009	1.49(1.06−2.09)	0.019
**Neutrophils** **	1.05(1.02–1.09)	0.004	1.05(1.02–1.09)	<0.001
**Severe** **COPD** ***	1.48(1.13–1.93)	0.004	1.41(1.06–1.86)	0.017
**Very Severe** **COPD** ***	1.97(1.45–2.67)	<0.001	2.19(1.58–3.02)	<0.001
**Azithromycin** **Vs.** **Placebo**	1.03(0.84–1.27)	0.75	0.97(0.78–1.20)	0.76
**BMI <20**	1.18(0.91–1.53)	0.20	1.30(0.99–1.70)	0.05
**Pack years >40** **years**	1.17(0.95–1.42)	0.14	1.27(1.02–1.70)	0.034

&ast; Estimated hazard ratio associated with an increment of one year.

&ast;&ast; Estimated hazard ratio associated with an increment of 1 * 10^9^ cells/L.

&ast;&ast;&ast; Moderate COPD (79–50 FEV1% predicted), Severe COPD (30–50 FEV1% predicted) [28] and Very Severe COPD (<30 FEV1% predicted).

**Figure 3:**
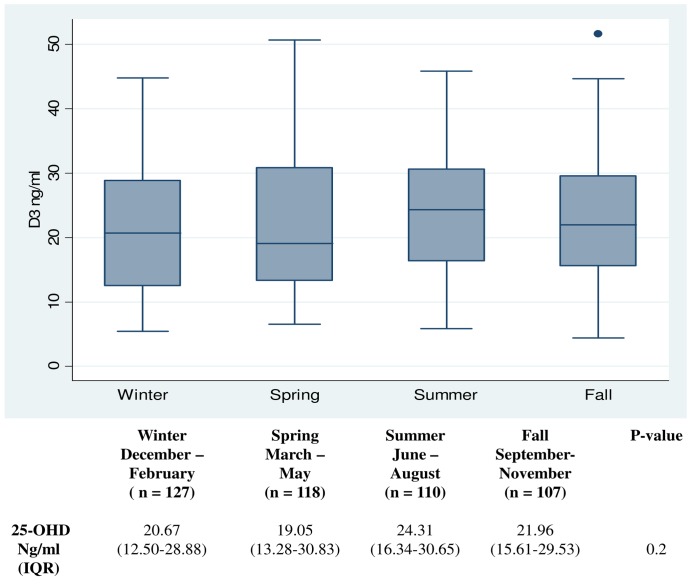
Seasonal differences in median serum 25-OHD levels. Significance levels were determined using Wilcoxon Rank sum test.

We also investigated whether there was any significant seasonal variation of 25-OHD serum levels in our patient group. The results are shown in figure 3. There was very little seasonal variation, and no significant differences.

**Figure 4:**
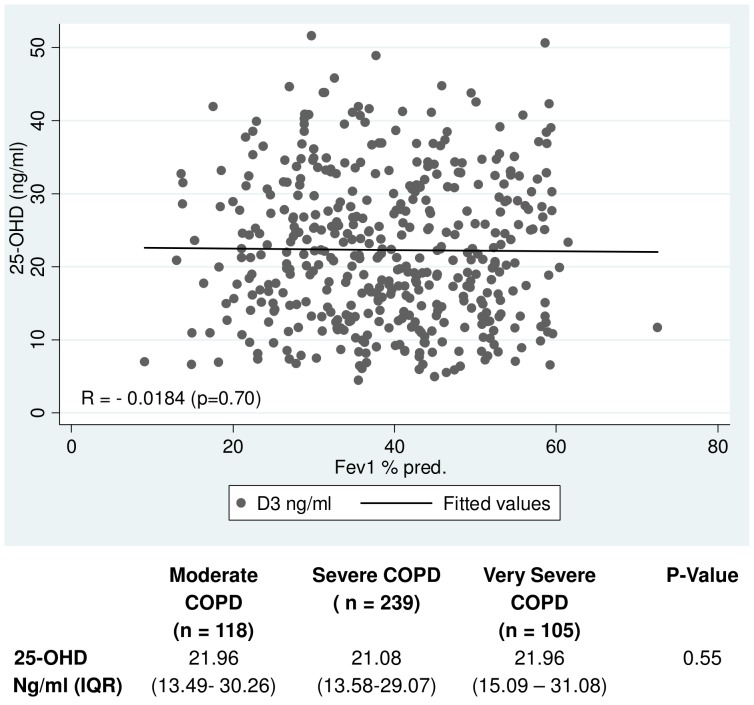
25-Hydroxyvitamin serum levels plotted as a function of Fev_1_ (% pred). Spearman coefficient was determined and the value noted in the lower left of the figure. Table below displays median 25-OHD levels distributed on COPD severity. Significance was tested using Kruskal Wallis equality-of-populations rank test.

Finally, we examined the serum level of 25-OHD in relation to pulmonary function. The results are shown in figure 4. Low 25-OHD serum levels were common as 348 of 462 patients were suffering from either insufficiency or deficiency (see table 1). We were however unable to demonstrate any significant difference in relation to COPD disease staging, and there was no relation between levels of 25-OHD and FEV1 (% pred.) (Figure 4).

## Discussion

We confirmed that 25-OHD serum deficiency and insufficiency is very common among patients with COPD [6], [24], i.e. 44.8% and 30.5% respectively. The main objective of our study was to examine whether there was an association between 25-OHD serum level and mortality. We did not find such an association. Increasing age was associated with increased mortality, as were COPD severity and Charlson scores >3. Likewise higher blood levels of neutrophils were associated with increased mortality as was a smoking history of more than 40 pack years at enrollment. We were unable to confirm correlations between disease severity and 25-OHD serum levels. In addition to this we did not find an association between low BMI and mortality. [24], [29].

Our finding of an association between increased mortality and higher blood neutrophile counts at baseline is interesting as neutrophil driven inflammation is believed to play an important role in the pathogenesis of COPD [30], [31]. Elevated levels of neutrophils could indicate a higher level of inflammation, which in turn could lead to a faster decline in lung function and ultimately a higher risk of early death. The patients in the present study were examined in stable phase of COPD. This reduces the likelihood that the observed correlation was caused by an episode of acute exacerbation or intercurrent infections. It is important however to emphasize that our observational finding does not prove a causal relationship between higher neutrophile counts and mortality.

In contrast to the study by Janssens et al. we did not find a correlation between severity of COPD and 25-OHD serum level [24]. In Jannsen’s study mean 25-OHD serum levels in patients in GOLD class II-IV fell from 20.35 ng/ml (n = 87) over 18.8 ng/ml (n = 75) and down to 16.0 ng/ml (n = 30). In comparison to this our results were consistently higher with mean values of 22.72 ng/ml (n = 118), 21.9 ng/ml (n = 239) and 23.1 ng/ml (n = 105). We used different assays in determining 25-OHD serum levels. We used liquid chromatography tandem mass spectrometry (LC-MS/MS). This method is very sensitive and considered the golden standard being able to detect very minute differences in 25-OHD serum levels [32]. In our study total CV was <7% from a level of 4.12 ng/ml, Calibration range 2.5–100 ng/ml.

It was surprising that we were unable to demonstrate a statistically significant effect of seasonality on vitamin D levels (figure 3). The measurements in our cohort were evenly distributed throughout the year. There was tendency toward higher levels during the summer months. One possible explanation could be that the patients followed in this cohort had fairly advanced COPD (median FEV1 38.5% pred), which may have limited the patients exposure to sunlight, the primary source of vitamin D. This may be especially true at Northern latitudes (i.e. 55 degrees N in this case) where it is well known that cutaneous synthesis of vitamin D is restricted to few months every year [33].Although seasonality has been demonstrated at these latitudes [34] we believe that decreased sun exposure during the period where most of the vitamin D is synthesized may make distinctions between seasons more difficult in this particular cohort. We did not find a statistical significant correlation between BMI and mortality, a correlation that has been shown in previous studies [29], [35]. We did however find a trend for such an association (table 2). We believe this reflects two things. Our study population included fewer patients. Furthermore the median BMI in our group was rather high leaving few persons at risk with BMI <20 (n = 93). The association between low BMI and mortality is stronger with progressively worse GOLD stages as demonstrated by Landbo et al. We did not have the number of patients to test this hypothesis within subgroups in our cohort [29].

We were not able to establish a correlation between 25-OHD serum level and mortality in our study. Our cohort was well characterized and follow up was 99.1% complete. In total 353 patients out of 462 died during the observation period. This is within the outcome event limits described by Peduzzi et al., suggesting 10 events per covariate examined when performing a Cox PH [36]. The population was characterized by a high degree of ethnic homogeneity (99.3% Western European descent) and vitamin D supplements were not common at the time samples were taken. Furthermore fortification of food products is not legal in Denmark and thus not a factor when assessing 25-OHD serum levels. Supplementation with vitamin D has become increasingly prevalent, and our study population may not be representative of patients suffering from moderate to very severe COPD today. The size of our cohort was fairly small compared to the size of the observational studies, which have found a significant association between mortality and vitamin D. However, these studies were usually done in large samples of the general population, and not in particular in patients with COPD [37], [38]. Concerning statistical power the smaller size of our cohort is somewhat offset by the very high rate of the primary outcome measure (76% mortality rate), the long study period, and the fact that follow-up was almost 100%. Our study was able to detect a coefficient difference of 0.3 (HR 1.35) with a power of 80%.We used Cox PH and adjusted for several factors that could potentially influence mortality in patients suffering from moderate to very severe COPD. The KM curves display three survival curves lying almost superimposed on each other and the log rank test did not show any significant difference. One could argue that the usage of the traditional classification of 25-OHD insufficiency and deficiency does not apply to its role in mortality. The established levels for deficiency and insufficiency are based on observations that describe25-OHD levels sufficient to maintain calcium homeostasis. Extrapolation of these observations to apply to mortality may not be appropriate. Because of this we also investigated the effect of baseline serum levels of 25-OHD distributed on tertiles. No difference in mortality was found when using this approach in uni- or multivariate analysis (data not shown).

Recently treatment with high dose vitamin D in patients with COPD who had 25-OHD serum levels below 10 ng/ml was shown to be associated with a decreased risk of developing exacerbations [27]. In addition to this, it has been shown that 25-OHD level below 10 ng/ml in elderly men is associated with low peak expiratory flow rate (PEFR). We performed a post-hoc analysis of patients with 25-OHD serum levels below 10 ng/ml. This yielded 50 patients at risk. Neither univariate [HR 1.24 (CI 95%: 0.89–1.72 p 0.2)], nor multivariate analysis [HR 1.32 (CI 95%: 0.95–1.85 p 0.1)] yielded a statistical significant association between very low serum 25-OHD concentration and mortality. On the other side the sample size was too small to discard the possibility of such an association. A large scale investigation is needed to exclude 25-OHD as a prognostic marker of mortality in this patient group. Finally we decided to test 25-OHD serum level as a continual variable and was unable to demonstrate any effect of 25-OHD concentration and mortality [HR 0.99 (95% CI: 0.98–1.00 p: 0.10)].

Our findings support published studies in which the investigators were unable to find a correlation between vitamin D and FEV_1_ decline [6], or between 25-OHD levels and exacerbation frequency [26]. A recent high dose vitamin D intervention study was unable to demonstrate an effect of vitamin D supplementation on mortality [27]. The study did however have few mortalities and the observation period did not extend beyond 1 year.

In conclusion vitamin D deficiency and insufficiency is highly prevalent in patients suffering from moderate to very severe COPD. 25-OHD serum level does not correlate with mortality in this patient group, suggesting no or only a minor role of vitamin D in disease progression in this patient population. From our data it is not possible exclude a potential role of vitamin D in less severe COPD.
